# Aquaporin-1 Protein Levels Elevated in Fresh Urine of Renal Cell Carcinoma Patients: Potential Use for Screening and Classification of Incidental Renal Lesions

**DOI:** 10.1155/2014/135649

**Published:** 2014-04-06

**Authors:** Shilpa Sreedharan, John A. Petros, Viraj A. Master, Kenneth Ogan, John G. Pattaras, David L. Roberts, Fei Lian, Rebecca S. Arnold

**Affiliations:** ^1^Department of Urology, Emory University, 1365 Clifton Road NE, Clinic B 4221, Atlanta, GA 30322, USA; ^2^Atlanta VA Medical Center, 1670 Clairmont Rd, Decatur, GA 30033, USA; ^3^Department of Medicine, Emory University, 1525 Clifton Road, 423 Atlanta, GA 30322, USA

## Abstract

*Introduction and Objectives*. There are over 65,000 new cases of renal cell carcinoma (RCC) each year, yet there is no effective clinical screening test for RCC. A single report claimed no overlap between urine levels of aquaporin-1 (AQP1) in patients with and without RCC (Mayo Clin Proc. 85:413, 2010). Here, we used archived and fresh RCC patient urine to validate this report. *Methods*. Archived RCC, fresh prenephrectomy RCC, and non-RCC negative control urines were processed for Western blot analysis. Urinary creatinine concentrations were quantified by the Jaffe reaction (Nephron 16:31, 1976). Precipitated protein was dissolved in 1x SDS for a final concentration of 2 **μ**g/µL creatinine. *Results*. Negative control and archived RCC patient urine failed to show any AQP1 protein by Western blot analysis. Fresh RCC patient urine is robustly positive for AQP1. There was no signal overlap between fresh RCC and negative control, making differentiation straightforward. *Conclusions*. Our data confirms that fresh urine of patients with RCC contains easily detectable AQP1 protein. However, archival specimens showed an absence of detectable AQP1 indistinguishable from negative control. These findings suggest that a clinically applicable diagnostic test for AQP1 in fresh urine may be useful for detecting RCC.

## 1. Introduction

Over 65,000 individuals are diagnosed with renal cell carcinoma (RCC) in the United States each year, and over 13,500 of these cases are fatal [1]. Both the incidence and detection rate of RCC have been increasing steadily since the 1970s [2]. The only treatment with meaningful cure rate is surgical excision, but results vary based on the stage at which the cancer is diagnosed. The asymptomatic nature of the early stages of renal cell cancers makes detection at a curable stage difficult, accomplished primarily with cost intensive cross-sectional imaging. Because of this, approximately 30% of cases are detected when the cancer is locally advanced or metastatic [3]. Most early stages of renal cancer are detected inadvertently during radiologic procedures such as computed tomography [4]. Differential diagnoses of renal masses are exceedingly difficult when utilizing radiological imaging. In fact, one study demonstrated that, when presented with radiologic images of either renal cysts or carcinomas, practiced radiologists misdiagnosed the masses in 50% of the cases [5]. In 30–40% of patients with symptomatic renal cancer, there are already metastases in the lymph nodes or other organs [1]. RCC is resistant to chemotherapy and metastatic disease has a five-year survival rate of 5% or less [6]. Early detection of these tumors has several benefits, including the option for minimally invasive surgery or ablation in addition to cure rates with higher durability. A tumor removed when confined to the renal capsule has a survival rate exceeding 70% [6]. The lack of a diagnostic biomarker for RCC presents a significant drawback in screening and clinical evaluation of incidentally discovered renal masses at a time amenable to surgical cure. Most evaluations depend on the apparent size and growth rate of the tumor.

Renal cancers are both genetically and phenotypically heterozygous for different histologic tumor types [7–10]. The most frequently occurring subtype is clear cell renal cell carcinoma, which accounts for 80% of all renal cancers and greater than 90% of metastases [7–10]. This particular subtype originates from the cells of the renal proximal tubule as does the papillary subtype both of which have been shown to positively correlate with increased urinary output of the protein aquaporin-1 or AQP1 [11, 12].

AQP1 is a water-transport protein found in the glomerular capillary endothelium and apical membrane of the proximal tubule in normal kidneys [13]. Expression array analysis and qRT-PCR have demonstrated increased expression of AQP1 in the urine of patients with different renal tumor subtypes but most significantly in clear cell and papillary renal cancers [11]. A significant linear correlation exists between AQP1 protein concentrations and tumor size in proximal tubule originating tumors compared to nonsurgical controls [12]. These elevated concentrations of AQP1 are implicated in the increase of the metastatic and migratory potential of these subtypes [14].

In a single report by the Morrissey group, urinary AQP1 protein concentrations in patients with renal masses undergoing nephrectomies were quantified and determined to be significantly elevated when compared to nonnephrectomy surgery controls. However, nonmalignant renal masses were not meaningfully evaluated in this study. The researchers concluded that, with a specificity of 100% and a sensitivity of 100%, urinary AQP1 is a good candidate for a diagnostic biomarker of renal cancers [12]. In an attempt to further validate this finding, we performed a confirmatory study involving archived and fresh urine samples from patients with histologically proven renal cell carcinomas and benign renal masses.

## 2. Methods

### 2.1. Patient Sample Collection

The protocol was approved by the Emory University Institutional Review Board, and all patients and volunteers gave written, informed consent to participate. Eleven archived RCC urine samples were obtained from Emory University's kidney satellite tissue bank. These samples were not treated with any protease inhibitors or other degradation-preventative measures before being stored in liquid nitrogen. Eleven urine samples hereby referred to as “fresh samples” were collected prospectively from RCC patients and obtained prenephrectomy from the operating room upon insertion of the Foley catheter. At least 15 mL of urine was collected per patient. Fresh control urine samples were obtained in the Emory Clinic. All fresh samples were deidentified prior to use and immediately processed (see urine preparation, Section 2.2) within two hours of collection to ensure optimal protein stability before being stored at −80°C. Tumor type, stage, grade, and size were determined from postoperative pathology reports and the Emory University kidney satellite tissue banking database.

### 2.2. Urine Preparation

Immediately after collection for fresh prenephrectomy and negative control specimens, urine was centrifuged for ten minutes at 1800 ×g before dividing into 1.5 mL aliquots, followed by thorough mixing with 1/5 of cOmplete, a protease inhibitor cocktail tablet (Roche Diagnostics, Indianapolis, IN, USA) per aliquot. To date, aliquots testing positive for AQP1 have been stable up to 1 year. Archived prenephrectomy specimens were thawed, and for all three specimen types, creatinine was quantified with the Jaffe reaction. Briefly, this method utilizes the reaction between creatinine and picric acid in an alkaline solution. The resulting colorimetric change is measured with a spectrophotometer [15]. The amount of urine corresponding to 200 *μ*g of creatinine was calculated and proteins from this amount were precipitated with 2 mL of ice cold acetone : methanol (1 : 1) and centrifuged for ten minutes at 1800 ×g. The acetone : methanol wash and centrifuge were repeated before dissolving precipitated proteins in 100 *μ*L of 1x sodium dodecyl sulfate buffer such that the resultant creatinine concentrations per sample were 2 *μ*g/µL.

### 2.3. Western Analysis

The precipitated proteins were mixed with *β*-mercaptoethanol, incubated in a boiling water bath for five minutes, and loaded onto precast Any-kD Tris-glycine gels (Bio-Rad Laboratories Inc., Hercules, CA, USA) for electrophoresis so that each well contained 60 *μ*g of creatinine per sample. Normalization to creatinine with urine stored in these conditions is the standard for urinary protein processing via western blotting [16]. Proteins were transferred onto PVDF membranes and blocked with 5% nonfat milk in PBS-Tween 20. Blocked membranes were incubated overnight with anti-AQP1 (H-55) (Santa Cruz Biotechnology Inc., Santa Cruz, CA, USA), diluted 1 : 500 in blocking buffer. After washing, the membranes were incubated with a 1 : 2000 dilution of goat anti-rabbit IgG HRP-conjugated antibody (Cell Signaling Technology, Danvers, MA, USA) for two hours at room temperature and visualized by chemiluminescence. AQP1 levels were semiquantified in arbitrary units using ImageJ software for area under the curve (AUC) analysis.

## 3. Results

**Figure 1:**
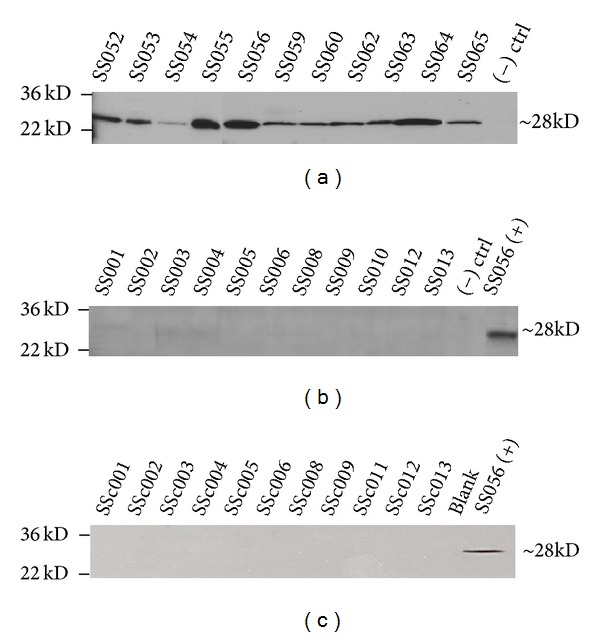
Western blot analysis of urinary AQP1 in RCC patients. (a) AQP1 in fresh urine collected from eleven patients suspected of RCC and undergoing nephrectomy or partial nephrectomy. The (–) control was protein isolated from the fresh urine of a patient with no known renal masses, malignant or benign, and no other known urological carcinomas. All samples were normalized to creatinine prior to electrophoresis. The reported molecular weight of AQP1 is 28 kD; L denotes the loading control ladder with which sample size was determined. Data are representative of 3 individual Western blots. (b) AQP1 in archived urine, prenephrectomy, of known clear cell renal cell carcinoma patients. (+) control sample is protein isolated from the fresh urine of patient SS056. (c) AQP1 in fresh urine collected from eleven volunteers with no evidence of renal disease, renal injury, or any other urological malignancy. Data are representative of 3 individual Western blots.

Western blot analysis yielded a band of 28 kD molecular weight for all eleven fresh urine samples from individuals with RCC and a single patient with oncocytoma, indicating the presence of AQP1 protein. This band was absent from archived urine samples and negative control urine, as depicted in Figure 1.

The tumor histology of patients providing fresh prenephrectomy urine for analysis is shown in Table 1. The eleven patients with positive AQP1 signals were determined to have clear cell renal cell carcinoma (*n* = 5), papillary renal cell carcinoma (*n* = 4), chromophobe RCC (*n* = 1), oncocytoma (*n* = 1), cystic nephroma (*n* = 1), and unclassified RCC (*n* = 1).

The patient tumor histologies for the eleven archived urine samples were obtained from the kidney satellite tissue banking database and are shown in Table 2. All eleven samples originated from patients with clear cell renal cell carcinoma (*n* = 11). The average tumor size for patients providing fresh urine samples was 4.73 cm compared to 7.79 cm in patients providing archived urine samples. Clinical characteristics, including age and gender, for control samples provided by healthy volunteers are depicted in Table 3.

The sensitivity and specificity of detecting fresh urinary AQP1 by Western blot analysis were both 100% for renal pathology, identical to previously reported figures by the Morrissey group. The clear distinction between fresh case and negative control indicates that both positive predictive value and negative predictive value were also 100%. The specificity and sensitivity for archived urine samples were not calculated due to results being indistinguishable from negative control samples.

**Figure 2:**
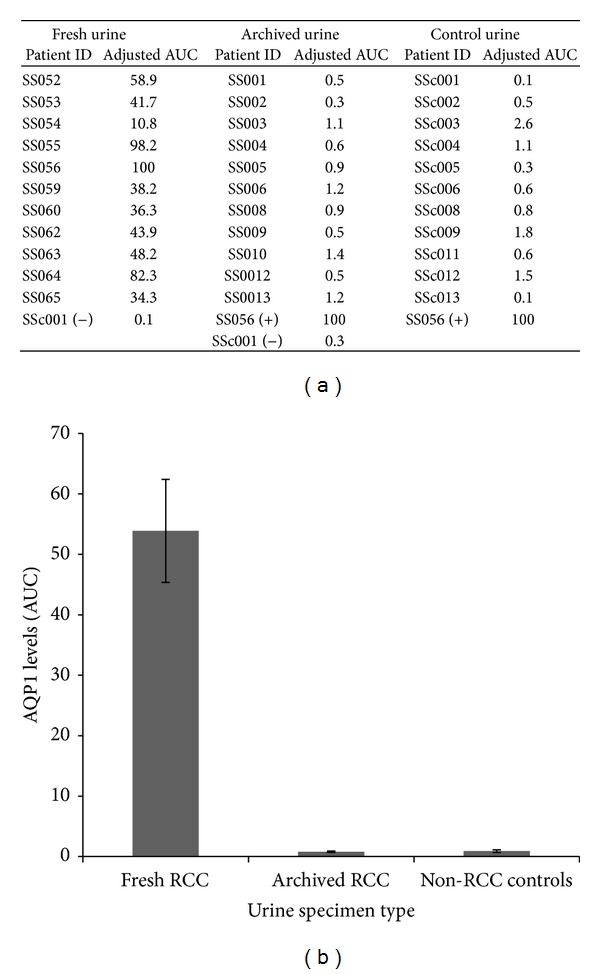
(a) Table containing arbitrary AUC used to quantify AQP1 levels across all three patients subsets, with each sample normalized to patient SS056. (b) Graphical depiction of the average semiquantitative AQP1 levels in patient fresh urine, archived urine, and volunteer control urine.

A graphical comparison of AQP1 levels of all three patient groups in arbitrary units derived from AUC analysis is shown in Figure 2. Fresh urine contained approximately 50 times the amount of detectable AQP1 than archived urine or fresh non-RCC (control) urine. These arbitrary numbers were derived by normalization against patient SS056 urinary AQP1 levels, which was used as the positive control in all experiments. Archived urine from known RCC cases and fresh urine from volunteers with no known urological malignancies had comparably low levels of AQP1 urinary protein.

## 4. Discussion

The presence of urinary AQP1 was not limited to patients with papillary and clear cell renal carcinomas in this study; patients with postoperative pathology reports of oncocytoma, benign cystic nephroma, and chromophobe renal cell carcinoma all had notable AQP1 bands via immunoblotting. For this reason, AQP1 may not be specific for solely malignant renal masses. Increased urinary levels of AQP1 were previously indicated to correlate with increased tumor size [12]. In radiologically detected masses that have no biopsy prior to surgery, the odds of malignancy increase by 16% with every 1 cm increase in tumor size [17]. Roughly 2% of patients with “benign” masses such as oncocytomas and cystic nephromas progress to metastatic disease in retrospective and prospective studies [18].

Urinary AQP1 protein levels could aid in diagnosis. Upon the radiologic finding of a suspicious renal mass, AQP1 could be utilized as a biomarker in conjunction with the scan to help determine likelihood that the mass is malignant. If detectable levels of AQP1 are lacking in the urine of these patients, patients may elect for active surveillance of the tumor in the place of a nephrectomy or partial nephrectomy, and this surveillance protocol could include periodic assays for urinary AQP1. Cystic renal masses have a growth rate of 0.09 cm/year while solid renal masses have a reported growth rate of 0.11 cm/year [19]. Given their slow progression, active surveillance is a viable management option for smaller or benign renal masses that are also negative for elevated urinary AQP1.

Perhaps the most important finding in this report is that, in all of the fresh urine from patients, malignant renal masses tested were robustly positive for AQP1 protein, while fresh controls were negative. This finding suggests that determination of urinary AQP1 levels could possibly be used for screening asymptomatic individuals. In this scenario, either the general population or subgroups at increased risk of developing RCC (positive family history, smokers, patients with genetic syndromes such as Von Hippel-Lindau disease, or other high risk groups such as hemodialysis patients) could benefit from periodic screening for AQP1 in the urine. Individuals with positive screens could then undergo workup through imaging, physical exam, and urinalysis. The ultimate clinical utility of RCC screening is to detect malignant lesions at an early stage when they could be effectively eliminated with minimal morbidity and higher cure rates.

Results of this experiment support the idea that urinary AQP1 is present and elevated in patients with kidney cancer and could therefore be useful in classifying incidentally discovered renal masses. However, there is an important distinction to be made: archived patient urine does not share this increased volume of AQP1 protein. The lack of a visible band at 28 kD in archived urine matched the negative control case and provided a clear distinction between it and fresh case samples. This could be due to lack of AQP1 in those archived urine samples selected. We speculate that, due to the absence of a standardized approach to prior collection and storage, the improper processing of the urine allowed for the degradation of the target protein before the assays were conducted. Improper storage may have allowed for protease activity to drastically decrease detectable amounts of AQP1. Any large cohort study done to confirm this data would require freshly acquired patient urine to be processed with low speed centrifugation, treated with a protease inhibitor, and stored in a −80°C freezer.

Further evaluation of AQP1 as a noninvasive kidney cancer biomarker should include larger cohorts, more quantitative measurements, and statistical analyses of not just clear cell and papillary RCCs but all renal masses, benign and malignant. A comparison of these findings with urine from patients with other urological cancers is a necessity. The completion of such studies could validate AQP1 as a clinically diagnostic tool and prevent unnecessary invasive surgeries in patients with benign renal lesions.

## 5. Conclusions

The sensitivity of urinary AQP1 for RCCs originating in the proximal tubule was corroborated with this confirmatory study. Fresh urine from patients with clear cell and papillary RCC subtypes exhibited increased concentrations compared to negative control urine; however, archived clear cell RCC urine specimens did not show detectable levels of AQP1 via immunoblotting. Urine from two patients with benign renal masses had detectable levels of AQP1, bringing to question the specificity of this protein as a potential biomarker; however, further quantitative studies are necessary to confirm this finding. Improperly processed archival urine samples are not recommended for further validation studies. The diagnostic potential of this protein in fresh clinical urine samples remains intact, and further studies involving the specificity of AQP1 are vital.

## Figures and Tables

**Table 1:** Tumor histology of patients providing fresh urine samples.

Patient ID	Patient gender	Patient age	Tumor histology*	Tumor size	Fuhrman grade	pTNM***
SS052	Male	52	RCC, clear cell	2.5 cm	2	pT1a, pNX, pMX
SS053	Female	62	RCC, clear cell	1.5 cm	2	pT1a, pNX, pMX
SS054	Male	61	RCC, papillary	5.5 cm	2	pT1b, pNX, pMX
SS055	Male	76	RCC, clear cell and papillary	1.0 cm, 0.4 cm**	2	pT1a, pNX, pMX
SS056	Male	60	RCC, clear cell and papillary	8.4 cm	3	pT2a, pNX, pMX
SS059	Male	72	RCC, papillary	10.6 cm, 1.2 cm**	3	pT2b, pNX, pMX
SS060	Male	74	RCC, clear cell	10.2 cm	3	pT3a, pN1, pMX
SS062	Female	53	RCC, unclassified	2.2 cm	3	pT1a, pNX, pMX
SS063	Male	42	RCC, chromophobe	8.0 cm	3	pT2a, pNX, pMX
SS064	Male	70	Oncocytoma	2.0 cm	n/a	n/a
SS065	Male	52	Cystic nephroma	8.0 cm	n/a	n/a

*RCC: renal cell carcinoma.

**Two measurements for the foci in a multicentric tumor.

*** pTNM: p: pathologic stage; T: primary tumor size; N: regional lymph node status; M: distant metastatic sites.

**Table 2:** Tumor histology of patients providing archived urine samples.

Patient ID	Patient gender	Patient age	Tumor histology*	Tumor size	Fuhrman grade	pTNM***
SS001	Male	58	RCC, clear cell	24.0 cm	3	pT3b, pN1, pMX
SS002	Male	68	RCC, clear cell	5.4 cm	4	pT1b, pNX, pMX
SS003	Male	64	RCC, clear cell	3.8 cm	2	pT3, pNX, pMX
SS004	Male	74	RCC, clear cell	4.5 cm	4	pT3a, pNX, pMX
SS005	Male	73	RCC, clear cell	5.0 cm	4	pT1, PNX, pM1
SS006	Male	56	RCC, clear cell	8.5 cm	3	pT3a, pNX, pMX
SS008	Female	63	RCC, clear cell	5.0 cm	4	pT3a, pNX, pMX
SS009	Female	75	RCC, clear cell	2.4 cm	2	pT3b, pNX, pMX
SS010	Female	69	RCC, clear cell	6.5 cm	4	pT1b, pNX, pMX
SS012	Female	64	RCC, clear cell	13.4 cm, 2.0 cm**	3	pT3, pNX, pM1
SS013	Male	61	RCC, clear cell	13.0 cm	3	pT3a, pN0, pM1

*RCC: renal cell carcinoma.

**Two measurements for the foci in a multicentric tumor.

*** pTNM: p: pathologic stage; T: primary tumor size; N: regional lymph node status; M: distant metastatic sites.

**Table 3:** Clinical characteristics of volunteers providing control urine samples.

Patient ID	Patient gender	Patient age
SSc001	Female	23
SSc002	Male	53
SSc003	Male	25
SSc004	Female	49
SSc005	Female	42
SSc006	Female	24
SSc008	Male	43
SSc009	Male	51
SSc011	Male	46
SSc012	Male	53
SSc013	Male	71
